# Supplementary material for: Defective BVES-mediated feedback control of cAMP in muscular dystrophy
Source: Nat Commun. 2023 Mar 30;14:1785. doi: 10.1038/s41467-023-37496-8 (PMC10063672; doi:10.1038/s41467-023-37496-8)
Supplement: Supplementary file 1 — Supplementary Information [file 41467_2023_37496_MOESM1_ESM.pdf]

Defective BVES-mediated feedback control of cAMP in muscular dystrophy

First author's surname: Li

Short title: Defective BVES-mediated cAMP signaling in muscular dystrophy

Haiwen Li[1, 2], Peipei Wang[1], Chen Zhang[1, 2], Yuanbojiao Zuo[1, 2], Yuan Zhou[1, 2], Renzhi Han[1, 2, *]

[1]Department of Pediatrics, Herman B Wells Center for Pediatric Research, Indiana University School of Medicine, Indianapolis, IN 46202, United States

[2]Department of Surgery, Davis Heart and Lung Research Institute, Biomedical Sciences Graduate Program, Biophysics Graduate Program, The Ohio State University Wexner Medical Center, Columbus, OH 43210, United States

*To whom correspondence should be addressed:
Renzhi Han, Ph.D.
Department of Pediatrics
Herman B Wells Center for Pediatric Research
Indiana University School of Medicine
Indianapolis, IN 46202
Phone: (317) 274-5642
E-mail: rh11@iu.edu

Supplementary Information

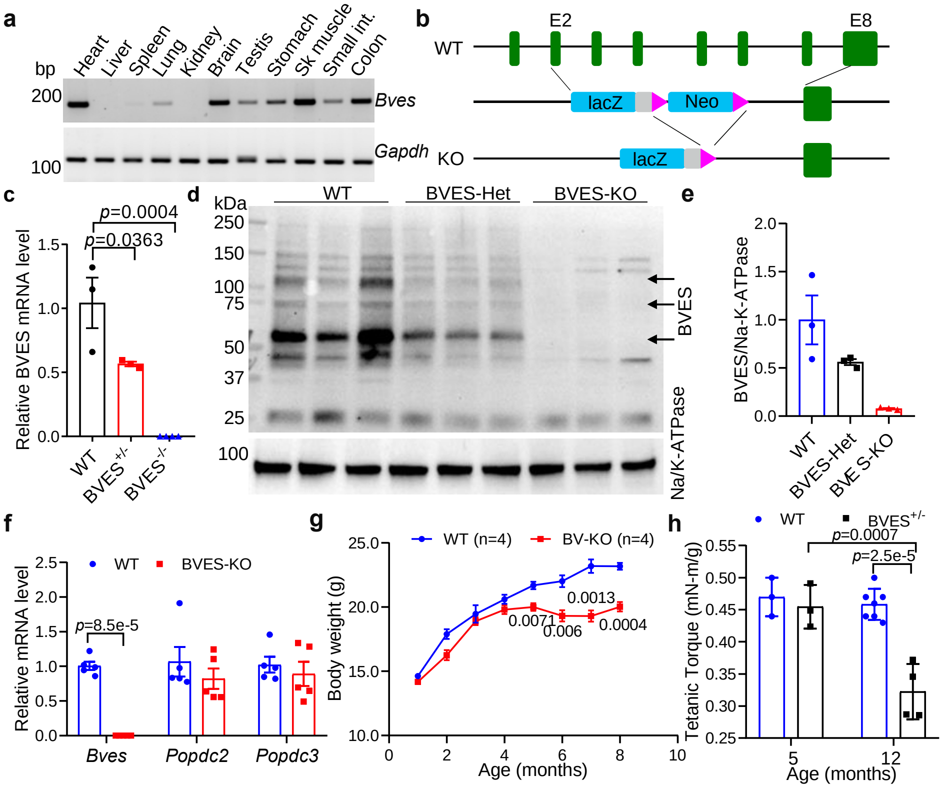

Supplementary Fig. 1: Development of BVES deficient mouse model and phenotype analysis. a, Expression of *Bves* in different mouse tissues examined by RT-PCR. (n=1 biologically independent experiments, each independent experiment are repeated twice). **b,** The diagram showing the strategy to generate BVES-KO mice. **c,** The expression of *Bves* in the skeletal muscle of WT (n=3), BVES-heterozygous (Het) (n=3) and BVES-KO (n=4) male mice examined by quantitative RT-PCR. one-way ANOVA with Turkey's multiple comparisons test. **d, e,** Western blot (**d**) and quantification (**e**) of BVES proteins in membrane extraction of WT(n=3), BVES-Het (n=3)and BVES-KO (n=3) male skeletal muscles. Na/K-ATPase was used as a loading control for membrane fractions. The arrows labeled the specific BVES bands. **f,** Expression of *Bves*, *Popdc2* and *Popdc3* in BVES-KO (n=5) and WT (n=5) male skeletal muscles by quantitative RT-PCR. Two-tailed unpaired Student's *t* test. **g,** Body weight gain of BVES-KO and age-matched WT female mice from one to eight months of age. Two-tailed paired Student's *t* test. **h,** Tetanic torque measurements of the posterior compartment muscles of WT (n=3 (5-month-old); n=7 (12-month-old) and BVES-Het (n=3 (5-month-old); n=4 (12-month-old)) mice at 5 and 12 months of age. Two-way ANONA with Tukey's multiple comparisons test. Data are mean ± SEM. Source data are provided as a Source Data file.

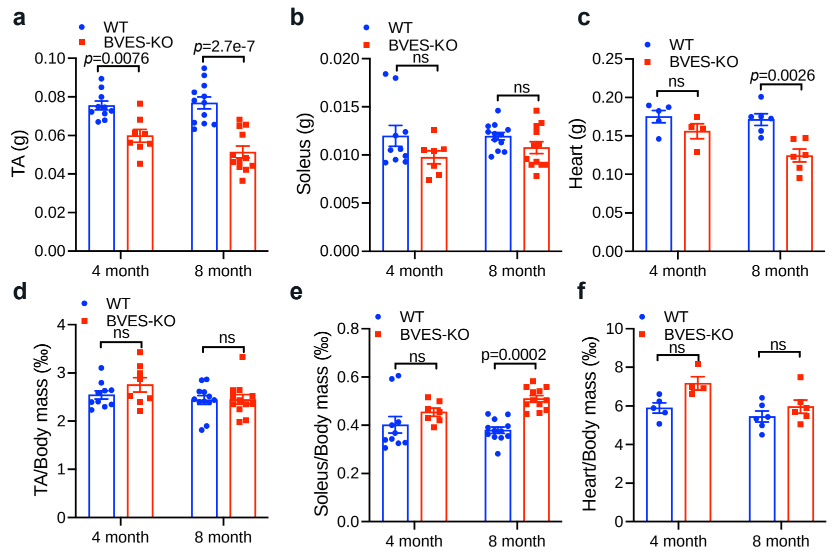

Supplementary Fig. 2: Muscle mass measurements in BVES-KO and WT male mice. a-c, The net mass of tibial anterior (TA) (**a**), soleus (**b**) and heart (**c**) in BVES-KO (n=8 (4-month-old); n=12 (8-month-old)) and WT (n=10 (4-month-old); n=12 (8-month-old)) mice at 4 and 8 months of age. **d-f,** The relative mass of TA (**d**), soleus (**e**) and heart (**f**) normalized to the body weight in BVES-KO and WT mice at 4 and 8 months of age. ns, not significant. Two-tailed unpaired Student's *t* test. Data are mean ± SEM. Source data are provided as a Source Data file.

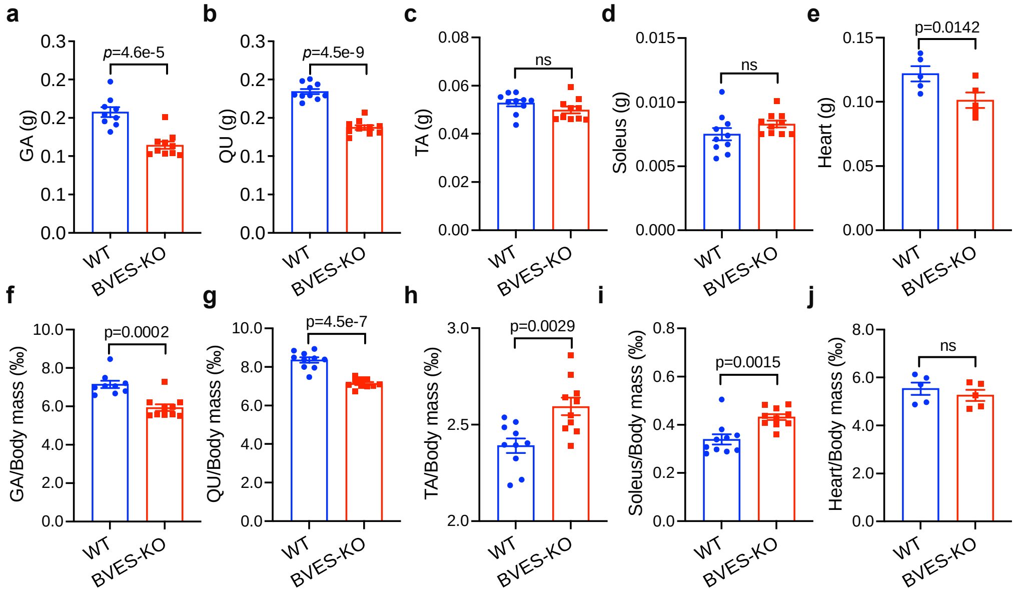

Supplementary Fig. 3: Muscle mass measurements in BVES-KO and WT female mice. a-e, The net mass of GA (**a**), QU (**b**), TA (**c**), soleus (**d**) and heart (**e**) in BVES-KO and WT female mice at 4 months of age. **f-j,** The relative mass of GA (**f**), QU (**g**), TA (**h**), soleus (**i**) and heart (**j**) normalized to the body weight in BVES-KO (muscle n=10) and WT (muscle n=10) female mice at 4 months of age. ns, not significant. Two-tailed unpaired Student's *t* test. Data are mean ± SEM. Source data are provided as a Source Data file.

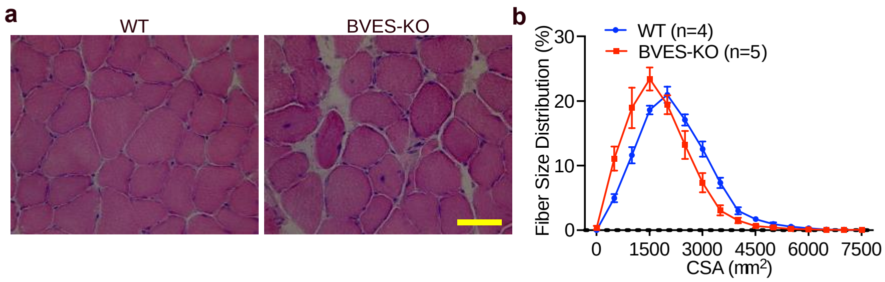

Supplementary Fig. 4: Histopathology analysis of GA muscles in 4-month-old BVES-KO and WT male mice. a, Representative H&E staining images of GA muscle cross-sections from BVES-KO and WT mice at 4 months of age. Scale bar: 100 μm. **b,** The CSA distribution of GA muscles from 4-month-old BVES-KO and WT mice. Data are mean ± SEM. Source data are provided as a Source Data file.

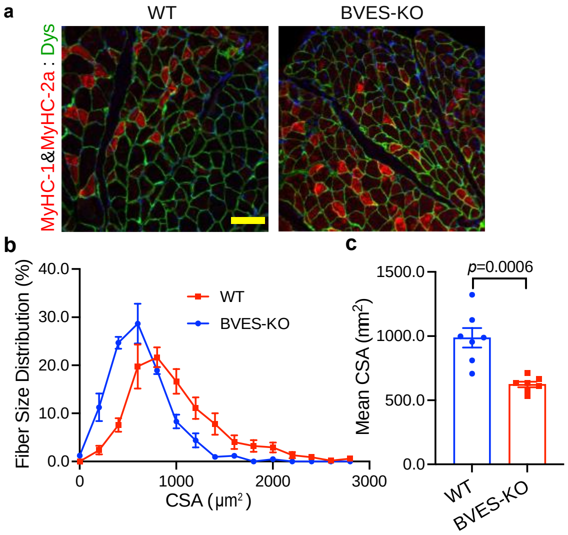

Supplementary Fig. 5: Histopathology of FDB muscle from 4-month-old BVES-KO and WT male mice. a, Representative immunofluorescence images of BVES-KO and WT FDB muscle sections stained with dystrophin (Dys, green) and the myosin heavy chain antibodies (MyHC-I and MyHC-IIa, red). Scale Bar: 100 μm. **b,** The CSA distribution of MyHC-x fibers in FDB muscles from 8-month-old BVES-KO (n=7) and WT (n=7) mice. CSA were quantified from the intact section areas of FDB muscles per mouse. **c**, Mean CSA of MyHC-x fibers in FDB muscles from 8-month-old BVES-KO (n=7) and WT (n=7) mice. Two-tailed unpaired Student's *t* test. Data are mean ± SEM. Source data are provided as a Source Data file.

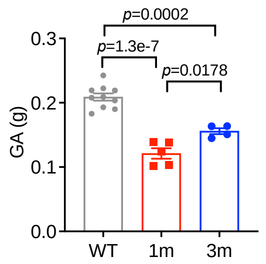

Supplementary Fig. 6: The net mass of AAV9-BVES treated GA muscles from BVES-KO male mice. WT (n=10, 4 months old, re-plotted from Fig. 2c), AAV9-BVES treated GA muscles at 1 (n=5) and 3 (n=4) months after injection from BVES-KO mice. One-way ANONA with Tukey's multiple comparisons test. Data are mean ± SEM. Source data are provided as a Source Data file.

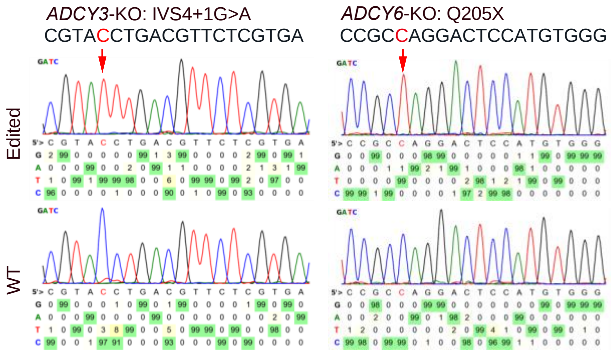

Supplementary Fig. 7: Generation of ADCY3/6 double KO HEK293 cell line with stable expression of cAMPr. The cAMPr stably transduced HEK293 cells were transfected with AncBE4max and the guide RNAs (gRNAs) targeting *ADCY3* and *ADCY6* to generate *ADCY3*_IVS4+1G>A and *ADCY6*_Q205X mutations. Sanger sequencing of single cell clone confirmed that this cell line carried the homozygous ADCY3 and ADCY6 mutations.

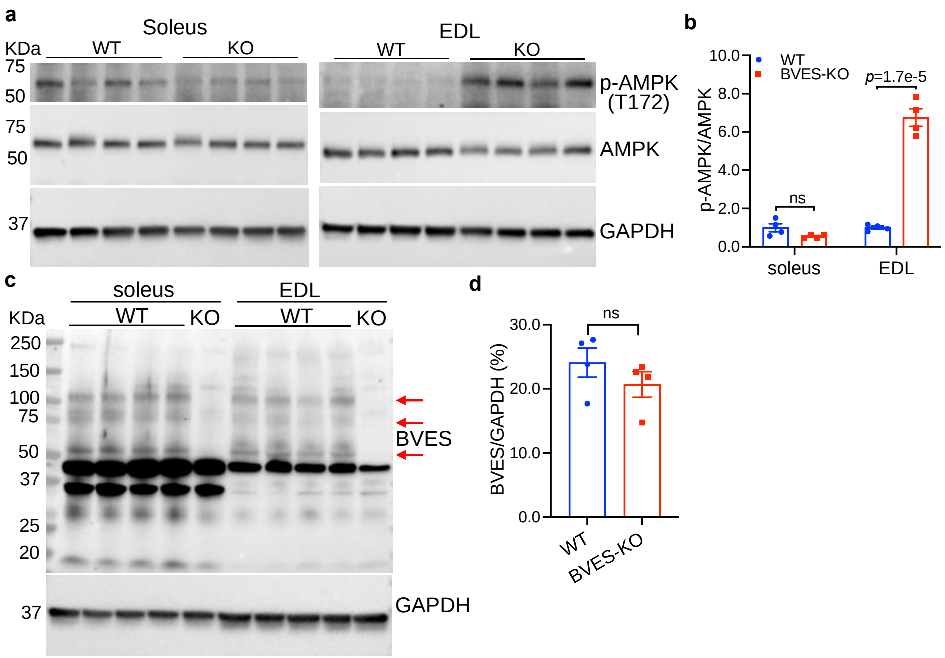

Supplementary Fig. 8: Western blot analysis of BVES and AMPK in soleus and EDL muscles. a, b, Western blot (**a**) and quantification (**b**) of AMPK and phosphorylated AMPK in soleus and EDL muscles from WT (n=4) and BVES-KO (n=4) male mice. **c, d,** Western blot (**c**) and quantification (**d**) of BVES in soleus (n=4) and EDL (n=4) muscles from WT mice. BVES-KO sample was loaded as a reference to identify the specific BVES bands (red arrows). ns, not significant. Two-tailed unpaired Student's *t* test. Data are mean ± SEM. Source data are provided as a Source Data file.

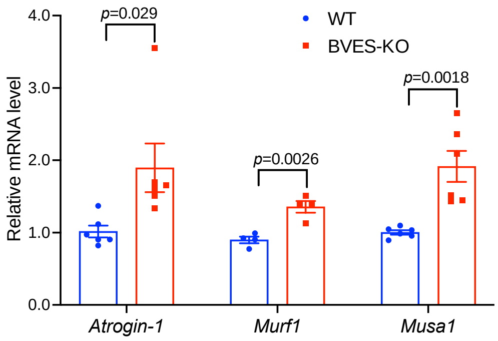

Supplementary Fig. 9: Expression of ubiquitination E3 ligases in skeletal muscle.

The transcript expression levels of *Atrogin-1*(WT: n=6; KO: n=6), *Murf1*(WT: n=4; KO: n=4) and *Musa1*(WT: n=6; KO: n=6) determined by quantitative RT-PCR. Two-tailed unpaired Student's *t* test. Data are mean ± SEM. Source data are provided as a Source Data file.

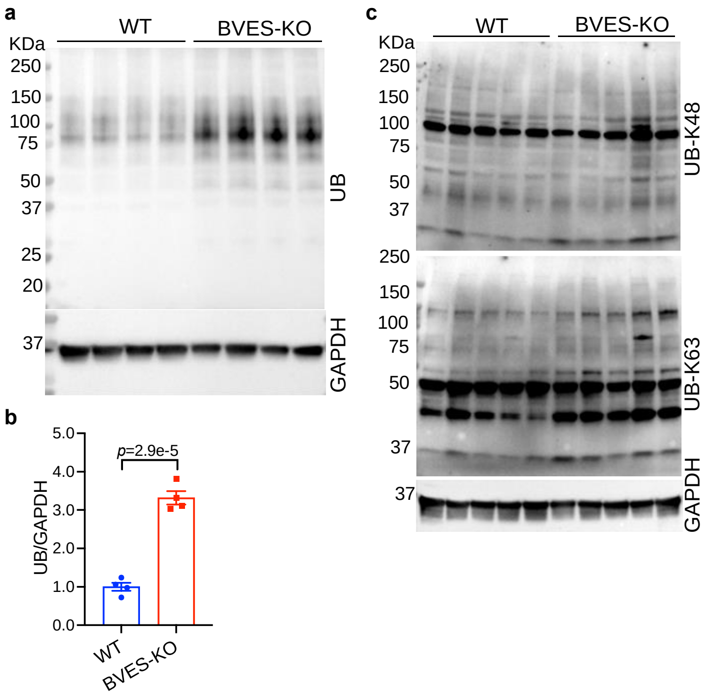

Supplementary Fig. 10: Western blot analysis of ubiquitination in BVES-KO muscle. a, b, Western blot (**a**) and quantification (**b**) of ubiquitination in total GA muscle extracts from 2-month-old WT (n=4) and BVES-KO (n=4) male mice. Two-tailed unpaired Student's *t* test. Data are mean ± SEM. **c,** Western blot of K48 and K63 ubiquitination in total GA muscle extracts from 8-month-old WT and BVES-KO male mice (n=2 biologically independent experiments, each independent experiment are repeated five times). Source data are provided as a Source Data file.

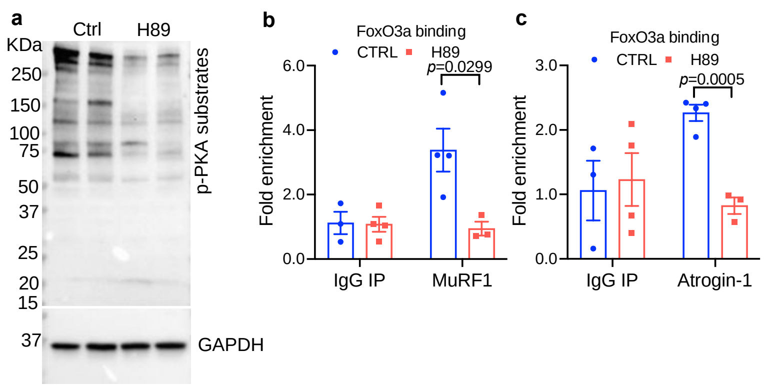

Supplementary Fig. 11: ChIP assay of FoxO3a activity in BVES-KO muscle in response to PKA inhibition. a, Western blot of p-PKA substrates in GA muscles from BVES-KO male mice without or with H89 treatment. **b, c,** ChIP assay showing the relative recruitment of FoxO3 on the promoters of selected atrophy-related genes. ChIP assays were performed in GA muscles from BVES-KO mice without or with H89 treatment. IgG was used as the reference. n=3 or 4 for each group. Two-tailed unpaired Student's *t* test. Data are mean ± SEM. Source data are provided as a Source Data file.

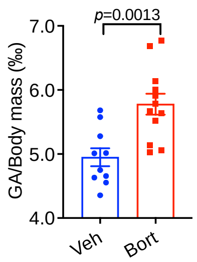

Supplementary Fig. 12: Improvement of muscle mass after bortezomib treatment.

The normalized GA muscle mass to body weight in BVES-KO male mice with vehicle (n=10) or bortezomib (n=12) treatment. Two-tailed unpaired Student's *t* test. Data are mean ± SEM. Source data are provided as a Source Data file.

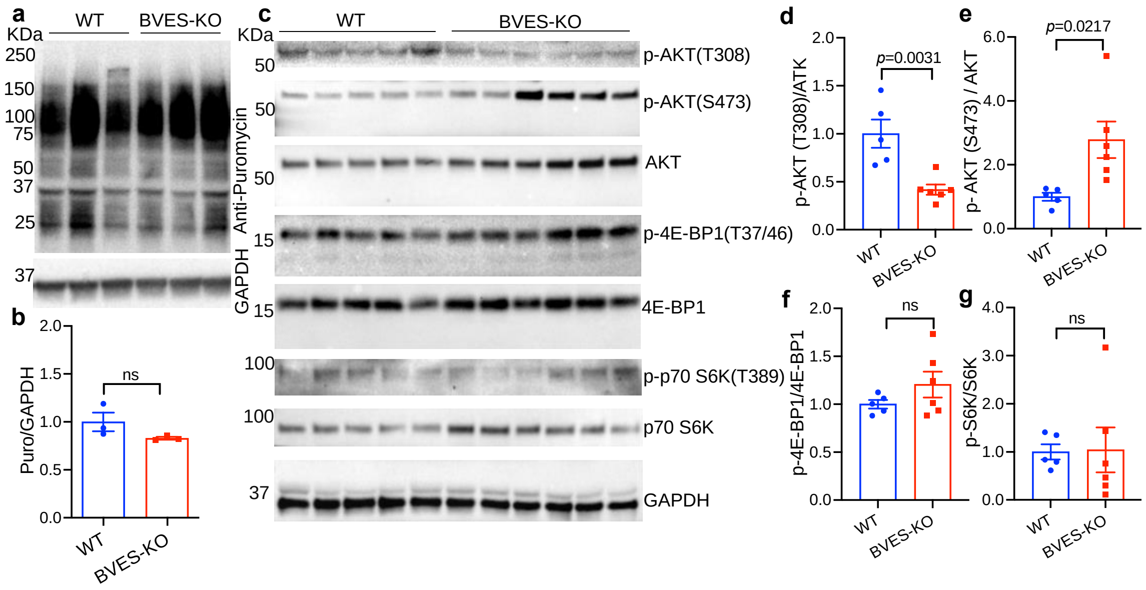

Supplementary Fig. 13: Unaffected protein biosynthesis in BVES-KO skeletal muscles. a, b, Western blot (**a**) and quantification(**b**) of the puromycin-labeled peptides in WT (n=3) and BVES-KO (n=3) male mice. ns, not significant. **c-g,** Western blot (**c**) and quantification of AKT signaling related with protein translation including p-AKT(T308) (**d**), p-AKT(S473) (**e**), p-4E-BP1(T37/46) (**f**) and p-p70 S6K(T389) in WT (n=5) and BVES-KO (n=6) male GA muscles. (**g**). ns, not significant. Two-tailed unpaired Student's *t* test. Data are mean ± SEM. Source data are provided as a Source Data file.

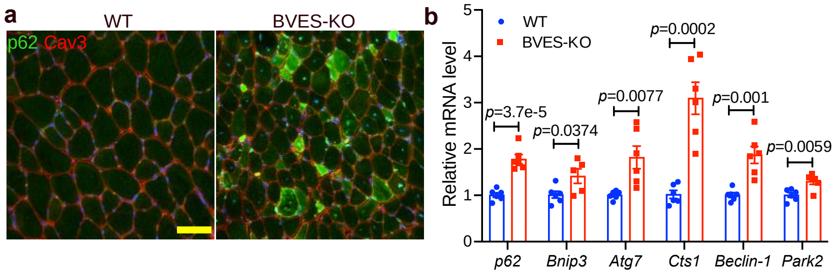

Supplementary Fig. 14: Alternation of autophagy in BVES-KO skeletal muscles. a, Immunostaining of p62 and Cav3 in GA muscles from 8-month-old WT and BVES-KO male mice. Scale bar: 50 µm. **b,** mRNA levels of the autophagy associated genes *p62*, *Bnip3, Atg7, Cts1, Becn1* and *Park2* determined by quantitative RT-PCR in WT (n=6) and BVES-KO (n=6) male GA muscles. Two-tailed unpaired Student's *t* test. Data are mean ± SEM. Source data are provided as a Source Data file.

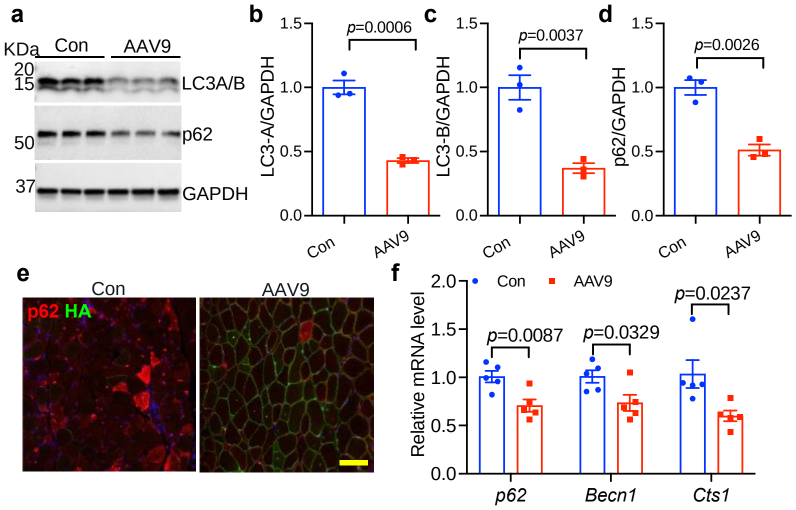

Supplementary Fig. 15: Improvement of autophagy defect in BVES-KO skeletal muscle following AAV9-BVES gene transfer. a-d, Western blot (**a**) of autophagy markers in control (n=3) and AAV9-BVES treated (n=3) GA muscles from BVES-KO male mice and quantification of LC3A (**b**), LC3B (**c**) and p62 (**d**) normalized by GAPDH. **e,** Immunostaining of p62 and BVES-HA in control and AAV9-BVES treated GA muscles from BVES-KO male mice. Scale bar: 50 µm. **f,** Quantitative RT-PCR analysis of the genes related with autophagy in control (n=5) and AAV9-BVES treated (n=5) GA muscles from BVES-KO male mice. Two-tailed unpaired Student's *t* test. Data are mean ± SEM. Source data are provided as a Source Data file.

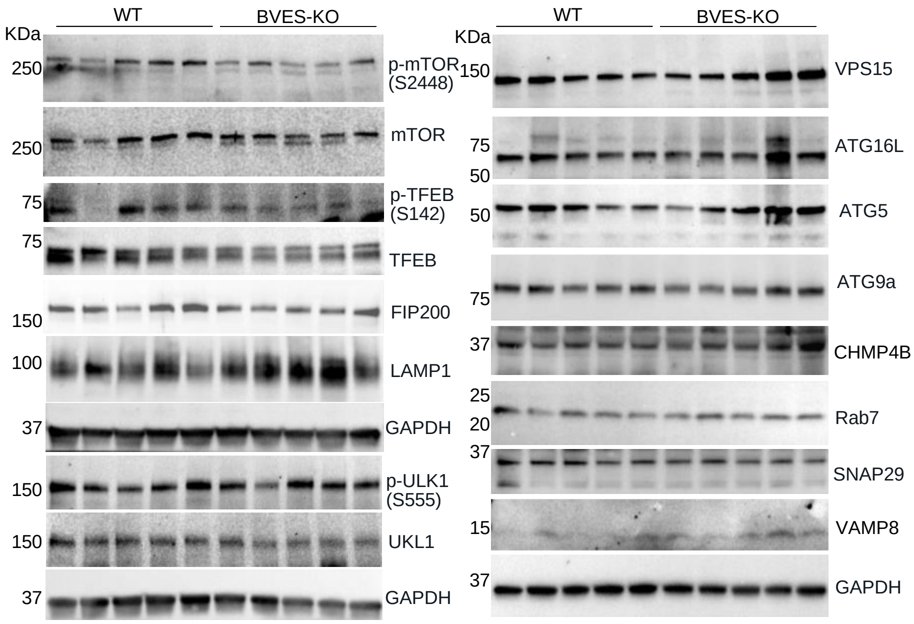

Supplementary Fig. 16: Expression of autophagy related proteins in BVES-KO skeletal muscles. Western blot analyses of autophagy related proteins in WT (n=5) and BVES-KO male (n=5) GA muscles. Source data are provided as a Source Data file.
