# Supplementary material for: Defective BVES-mediated feedback control of cAMP in muscular dystrophy
Source: Nat Commun. 2023 Mar 30;14:1785. doi: 10.1038/s41467-023-37496-8 (PMC10063672; doi:10.1038/s41467-023-37496-8)
Supplement: Supplementary file 3 — Description of Additional Supplementary Files [file 41467_2023_37496_MOESM3_ESM.pdf]

Description of Additional Supplementary Files

File name: Supplementary Data 1

Description: List of identified proteins from the IP-MS study.

File name: Supplementary Data 2

Description: List of primers used in this study.

File name: Supplementary Data 3

Description: List of antibodies used in this study.
