# Supplementary material for: Defective BVES-mediated feedback control of cAMP in muscular dystrophy
Source: Nat Commun. 2023 Mar 30;14:1785. doi: 10.1038/s41467-023-37496-8 (PMC10063672; doi:10.1038/s41467-023-37496-8)
Supplement: Supplementary file 7 — Reporting Summary [file 41467_2023_37496_MOESM7_ESM.pdf]

nature portfolio

Corresponding author(s): Renzhi Han

Last updated by author(s): Mar 3, 2023

Reporting Summary

Nature Portfolio wishes to improve the reproducibility of the work that we publish. This form provides structure for consistency and transparency in reporting. For further information on Nature Portfolio policies, see our Editorial Policies and the Editorial Policy Checklist.

Statistics

For all statistical analyses, confirm that the following items are present in the figure legend, table legend, main text, or Methods section.

n/a	Confirmed	
☐	☒	The exact sample size (n) for each experimental group/condition, given as a discrete number and unit of measurement
☐	☒	A statement on whether measurements were taken from distinct samples or whether the same sample was measured repeatedly
☐	☒	The statistical test(s) used AND whether they are one- or two-sided<br>*Only common tests should be described solely by name; describe more complex techniques in the Methods section.*
☒	☐	A description of all covariates tested
☐	☒	A description of any assumptions or corrections, such as tests of normality and adjustment for multiple comparisons
☐	☒	A full description of the statistical parameters including central tendency (e.g. means) or other basic estimates (e.g. regression coefficient) AND variation (e.g. standard deviation) or associated estimates of uncertainty (e.g. confidence intervals)
☐	☒	For null hypothesis testing, the test statistic (e.g. F, t, r) with confidence intervals, effect sizes, degrees of freedom and P value noted<br>*Give P values as exact values whenever suitable.*
☒	☐	For Bayesian analysis, information on the choice of priors and Markov chain Monte Carlo settings
☒	☐	For hierarchical and complex designs, identification of the appropriate level for tests and full reporting of outcomes
☒	☐	Estimates of effect sizes (e.g. Cohen's d, Pearson's r), indicating how they were calculated

Our web collection on statistics for biologists contains articles on many of the points above.

Software and code

Policy information about availability of computer code

Data collection	ZEN 2011 software was used to collect confocal fluorescence images; INFINITY CAPTURE software v6.5.0 was used for H&E image; NIS-Elements AR version 4.50 was used to record epifluorescence image; ChemiDoc XRS+ system was used to collect Western blot data; QuantStudio Design &Analysis software v1.5.1 was used to collect realtime PCR data; DMA v5.501 was used to collect muscle contractility data; SeDaCOM v2.0 was used for recording running distance and time.
Data analysis	Sample size was estimated with G-power software 3.1. Graphpad prism 8.0.1 was used to analyze the data. Adobe Illustrator 27.3.1 was used to assemble figures. Western blots were imaged and quantified using Image Lab 5.2.1 software. Sanger sequencing trace data were analyzed by using BEAT vl.0, which is published and available at https://github.com/Hanlab-OSU/Beat. Mass spectrum Data were searched against mouse database on MASCOT Via PD (ProteomeDiscoverer), and then analyzed using Scaffold 5.2.2. Fiber size and CNF quantification were carried out using Myosight program with manual calibration (available at https://github.com/LyleBabcock/MyoSight/tree/master).

For manuscripts utilizing custom algorithms or software that are central to the research but not yet described in published literature, software must be made available to editors and reviewers. We strongly encourage code deposition in a community repository (e.g. GitHub). See the Nature Portfolio guidelines for submitting code & software for further information.

Data

Policy information about availability of data

All manuscripts must include a data availability statement. This statement should provide the following information, where applicable:

- Accession codes, unique identifiers, or web links for publicly available datasets
- A description of any restrictions on data availability
- For clinical datasets or third party data, please ensure that the statement adheres to our policy

The IP-MS data have been deposited in PRoteomics IDEntifications Database (https://www.ebi.ac.uk/pride/) under the accession number "PXD036346 [https://www.ebi.ac.uk/pride/archive?keyword=PXD036346]". All data generated or analyzed during this study are included within the article and its Supplementary Information files. Source data are provided with this paper.

Human research participants

Policy information about studies involving human research participants and Sex and Gender in Research.

Reporting on sex and gender	not applicable
Population characteristics	not applicable
Recruitment	not applicable
Ethics oversight	not applicable

Note that full information on the approval of the study protocol must also be provided in the manuscript.

Field-specific reporting

Please select the one below that is the best fit for your research. If you are not sure, read the appropriate sections before making your selection.

☒ Life sciences ☐ Behavioural & social sciences ☐ Ecological, evolutionary & environmental sciences

For a reference copy of the document with all sections, see nature.com/documents/nr-reporting-summary-flat.pdf

Life sciences study design

All studies must disclose on these points even when the disclosure is negative.

Sample size	Sample size is estimated with G-power software 3.1 by the significance level (0.05), effect size (based on the data in our previous studies) and a given power (usually 0.8). We also conducted power analysis after the experiment and found enough power value (>0.8) in each statistical analysis.
Data exclusions	No data exclusion
Replication	All the experimental findings were repeated with a minimum of three independent experiments (or animals). All attempts for replication were successful.
Randomization	Mice and cells were assigned randomly into experimental groups and processed in an arbitrary order.
Blinding	All the enrolled mice or subsequent samples were labeled only with mouse ID numbers without genotype or type of treatment information. Genotype or treatment type were decoded after the data acquisition and quantification analysis were complete. The culture studies (transfection, DNA isolation, PCR, sequencing, live cell imaging, co-IP) were carried out identically through standard procedures that should not bias outcomes.

Reporting for specific materials, systems and methods

We require information from authors about some types of materials, experimental systems and methods used in many studies. Here, indicate whether each material, system or method listed is relevant to your study. If you are not sure if a list item applies to your research, read the appropriate section before selecting a response.

Materials & experimental systems

n/a	Involved in the study
☐	☒ Antibodies
☐	☒ Eukaryotic cell lines
☒	☐ Palaeontology and archaeology
☐	☒ Animals and other organisms
☒	☐ Clinical data
☒	☐ Dual use research of concern

Methods

n/a	Involved in the study
☒	☐ ChIP-seq
☒	☐ Flow cytometry
☒	☐ MRI-based neuroimaging

Antibodies

Antibodies used

Rabbit anti-phospho-AMPK (Thr172) Cell Signaling Technology #2535 WB(l:1000);
Rabbit anti-AM PK Cell Signaling Technology #5831 WB(l:1000);
Rabbit anti-FoxOl Cell Signaling Technology #2880 WB(l:1000);
Rabbit anti-Fox03 Cell Signaling Technology #2497 WB(l:1000), IF(l:100), CHIP (1:200);
Rabbit anti-Atrogin-1 ECM Biosciences #AP2041 WB(l:500);
Rabbit anti-MuRFl ((-terminal region) ECM Biosciences #MP3401 WB(l:500);
Rabbit anti-LKBl Cell Signaling Technology #3047 WB(l:1000);
Rabbit anti-phospho-LKBl (Ser428) Cell Signaling Technology #3482 WB(l:1000);
Rabbit anti-ULKl Cell Signaling Technology #8054 WB(l:1000);
Rabbit anti-phospho-ULKl (Ser555) Cell Signaling Technology #5869 WB(l:1000);
Rabbit anti-GAPDH Cell Signaling Technology #5174 WB(l:1000);
Rabbit anti-p62 Cell Signaling Technology #5114 WB(l:1000);
Rabbit anti-p62 Proteintech #18420-1-AP IF(l:100);
Rabbit anti-BVES Sigma-Aldrich #HPA014788 WB(l:500), IF(l:50);
Rabbit anti-HA Cell Signaling Technology #3724 WB(l:1000), IF(l:200);
Rabbit anti-Phospho-PKA Substrate (RRXS*/T*) Cell Signaling Technology #9624 WB(l:1000);
Rabbit anti-LC3A/B Cell Signaling Technology #4108 WB(l:1000);
Rabbit anti-FIP200 Cell Signaling Technology #12436 WB(l:1000);
Rabbit anti-VAMP7 Proteintech #22268-1-AP WB(l:500);
Mouse anti-Ubiquitin Santa Cruz #sc-8017 WB(l:500);
Rabbit K48-linkage Specific Polyubiquitin (D9D5) Cell Signaling Technology #8081 WB(l:500)
Rabbit K63-linkage Specific Polyubiquitin (D7All) Cell Signaling Technology #5621 WB(l:500)
Rabbit anti-phospho-4E-BP1 (Thr37 /46) Cell Signaling Technology #2855 WB(l:1000);
Rabbit anti-4E-BP1 Cell Signaling Technology #9644 WB(l:1000);
Rabbit anti-AKT Cell Signaling Technology #9272 WB(l:1000);
Rabbit anti-phospho-Akt (Ser473) Cell Signaling Technology #4060 WB(l:1000);
Rabbit anti-phospho-Akt (Thr308) Cell Signaling Technology #9275 WB(l:500);
Rabbit anti-phospho-p70 S6 Kinase (Thr389) Cell Signaling Technology #9205 WB(l:500);
Rabbit anti-p70 S6 Kinase Cell Signaling Technology #9202 WB(l:1000);
Rabbit anti-Na,K-ATPase Cell Signaling Technology #3010 WB(l:1000);
Rabbit anti-dystrophin Spring Bioscience #E2660 IF(l:200);
Mouse anti-MyHC-lla (SC-71) Developmental Studies Hybridoma Bank #AB_2147165 IF(l:50);
Mouse anti-MyHC-llb ((10F5) Developmental Studies Hybridoma Bank #AB_1157896 IF(l:50);
Mouse anti-MyHC-I (BA-D5) Developmental Studies Hybridoma Bank #AB_2235587 IF(l:50);
Mouse Anti-Caveolin 3 BD #610420 IF(l:400);
mouse anti-puromycin clone 12D10 Sigma-Aldrich #MABE343 WB(l:1000);
Rabbit anti-ATG16L Cell Signaling Technology #8089 WB(l:1000);
Rabbit anti-ATG5 Cell Signaling Technology #12994 WB(l:1000);
Rabbit anti-ATG9a Cell Signaling Technology #13509 WB(l:1000);
Rabbit anti-VPS34 Cell Signaling Technology #4263 WB(l:1000);
Rabbit anti-VPS15 Cell Signaling Technology #14580 WB(l:1000);
Rabbit anti-Rab7 Cell Signaling Technology #9367 WB(l:1000);
Rabbit anti-LAM Pl Cell Signaling Technology #9091 WB(l:1000);
Rabbit anti-STX17 Proteintech #17815-1-AP WB(l:500);
Rabbit anti-mTOR (7C10) Cell Signaling Technology #2983 WB(l:500);
Rabbit anti-phospho-mTOR (Ser2448) Cell Signaling Technology #2971 WB(l:500);
Rabbit anti-SNAP29 Proteintech #12704-1-AP WB(l:1000);
Rabbit anti-phospho-TFEB(Ser142) Sigma-Aldrich #ABE1971 WB(l:500);
Rabbit anti-TFEB Cell Signaling Technology #4240 WB(l:1000);
Mouse anti-Flag M2 Sigma-Aldrich #F3165 WB(l:1000), IP (1:1000);
Rabbit anti-VAMPS Cell Signaling Technology #13060 WB(l:500);
Goat anti-Mouse lgG (H+L) Alexa Fluor'M 594 lnvitrogen#A-11032 IF(l:400);
Goat anti-Rabbit lgG (H+L) Alexa Fluor'M 488 lnvitrogen#A-11008 IF(l:400);
Donkey anti-Rabbit lgG (H+L) Alexa Fluor'M 568 lnvitrogen#A-10042 IF(l:400);
Secondary HRP-conjugated goat anti-mouse Cell Signaling Technology#7076 WB(l:4000);
Secondary HRP-conjugated goat anti-rabbit Cell Signaling Technology# 7074 WB(l:4000).
I

Validation

All antibodies are available on the manufacturer's websites as listed below. The antibodies have been validated by the

Validation

manufacturers. No additional validation was carried out.

1. Phospho-AMPKα (Thr172) (40H9) Rabbit mAb #2535:
The species: H M R Hm Mk Dm Sc
Application: WB/IP/IHC
Website: https://www.cellsignal.com/products/primary-antibodies/phospho-ampka-thr172-40h9-rabbit-mab/2535

2. AMPKα (D5A2) Rabbit mAb #5831
The species: H M R Mk B
Application: WB/IP
Website: https://www.cellsignal.com/products/primary-antibodies/ampka-d5a2-rabbit-mab/5831

3. FoxO1 (C29H4) Rabbit mAb #2880
The species: H M R Mk
Application: WB/IP/IHC/IF/F/CHIP
Website: https://www.cellsignal.com/products/primary-antibodies/foxo1-c29h4-rabbit-mab/2880

4. FoxO3a (75D8) Rabbit mAb #2497
The species: Human/House mouse/Rat/Guinea Pig
Application: WB/IF /IHC/ICC/CHIP/Ab Array/FACS/ICC/ICC-IF/IHC-IF
Website: https://www.citeab.com/antibodies/123466-2497-foxo3a-75d8-rabbit-mab?des=37695e304dc8574d

5. Rabbit anti-Atrogin-1 ECM Biosciences #AP2041
The species: M R
Application: WB/IHC /ELISA
Website: https://ecmbio.com/products/ap2041

6. Rabbit anti-MuRFl ((-terminal region) ECM Biosciences #MP3401
The species: M R
Application: WB/ICC /ELISA
Website: https://ecmbio.com/products/mp3401

7. LKB1 (D60C5) Rabbit mAb #3047
The species: H M R Mk
Application: WB
Website: https://www.cellsignal.com/products/primary-antibodies/lkb1-d60c5-rabbit-mab/3047

8. Phospho-LKB1 (Ser428) (C67A3) Rabbit mAb #3482
The species: H M R Mk
Application: WB
Website: https://www.cellsignal.com/products/primary-antibodies/phospho-lkb1-ser428-c67a3-rabbit-mab/3482

9. ULK1 (D8H5) Rabbit mAb #8054
The species: H M R Mk
Application: WB/IP
Website: https://www.cellsignal.com/products/primary-antibodies/ulk1-d8h5-rabbit-mab/8054

10. Phospho-ULK1 (Ser555) (D1H4) Rabbit mAb #5869
The species: H M
Application: WB/IP
Website: https://www.cellsignal.com/products/primary-antibodies/phospho-ulk1-ser555-d1h4-rabbit-mab/5869

11. Phospho-p70 S6 Kinase (Thr389) Antibody #9205
The species: H M R Mk
Application: WB
Website: https://www.cellsignal.com/products/primary-antibodies/phospho-p70-s6-kinase-thr389-antibody/9205

12. GAPDH (D16H11) XP® Rabbit mAb #5174
The species: H M R Mk
Application: WB/IHC/IF
Website: https://www.cellsignal.com/products/primary-antibodies/gapdh-d16h11-xp-rabbit-mab/5174

13. SQSTM1/p62 Antibody #5114
The species: H M R Mk
Application: WB
Website: https://www.cellsignal.com/products/primary-antibodies/sqstm1-p62-antibody/5114

14. Rabbit anti-p62 Proteintech #18420-1-AP
The species: Human, Mouse, Rat, others
Application: WB, IP, IHC, IF, FC, CHIP, Others
Website: https://www.citeab.com/antibodies/980164-18420-1-ap-p62-sqstm1-antibody?des=fab1f65019015be0

15. Rabbit anti-BVES Sigma-Aldrich #HPA014788
The species: Human, mouse
Application: WB, IF, IHC
Website: https://www.citeab.com/antibodies/2304656-hpa014788-anti-bves-antibody-produced-in-rabbit?des=c6c911b56e3d10ba

Reference 1: Haiwen Li et al. BVES is a novel interactor of ANO5 and regulates myoblast differentiation, Cell & bioscience, 11(1), 222-222 (2021-12-30)
Reference 2: Haiwen Li et al. Systemic AAV9.BVES delivery ameliorates muscular dystrophy in a mouse model of LGMDR25, Mol Ther, 2023 Feb 1;31(2):398-408

16. HA-Tag (C29F4) Rabbit mAb #3724
The species: All
Application: WB/IP/IHC/IF/F/CHIP
Website: https://www.cellsignal.com/products/primary-antibodies/ha-tag-c29f4-rabbit-mab/3724

17. Phospho-PKA Substrate (RRXS*/T*) (100G7E) Rabbit mAb #9624
The species: All
Application: WB/IP
Website: https://www.cellsignal.com/products/primary-antibodies/phospho-pka-substrate-rrxs-t-100g7e-rabbit-mab/9624

18. LC3A/B Antibody #4108
The species: H M R
Application: WB/IF/F
Website: https://www.cellsignal.com/products/primary-antibodies/lc3a-b-antibody/4108

19. FIP200 (D10D11) Rabbit mAb #12436
The species: H M
Application: WB/IP
Website: https://www.cellsignal.com/products/primary-antibodies/fip200-d10d11-rabbit-mab/12436

20. VAMP7/TI-VAMP Polyclonal antibody
The species: Human, Mouse, Rat
Application: WB, IP, IHC, IF, ELISA
Website: https://www.ptglab.com/products/VAMP7-Antibody-22268-1-AP.htm

21. Mouse anti-Ubiquitin Santa Cruz #sc-8017
The species: Human, Mouse, Rat
Application: WB, IP, IHC, IF, ELISA, others
Website: https://www.citeab.com/antibodies/828150-sc-8017-ubiquitin-antibody-p4d1?des=3ba2961761f74301

22. K48-linkage Specific Polyubiquitin (D9D5) Rabbit mAb #8081
The species: All
Application: WB
Website: https://www.cellsignal.com/products/primary-antibodies/k48-linkage-specific-polyubiquitin-d9d5-rabbit-mab/8081

23. K63-linkage Specific Polyubiquitin (D7A11) Rabbit mAb #5621
The species: All
Application: WB
Website: https://www.cellsignal.com/products/primary-antibodies/k63-linkage-specific-polyubiquitin-d7a11-rabbit-mab/5621

24. Phospho-4E-BP1 (Thr37/46) (236B4) Rabbit mAb #2855
The species: H M R Mk Dm
Application: WB/IHC/IF/F
Website: https://www.cellsignal.com/products/primary-antibodies/phospho-4e-bp1-thr37-46-236b4-rabbit-mab/2855

25. Phospho-4E-BP1 (Thr37/46) (236B4) Rabbit mAb #2855
The species: H M R Mk
Application: WB/IP/IHC/IF/F
Website: https://www.cellsignal.com/products/primary-antibodies/4e-bp1-53h11-rabbit-mab/9644

26. Akt Antibody #9272
The species: H M R Hm Mk C Dm B Dg Pg GP
Application: WB/IP/IF/F
Website: https://www.cellsignal.com/products/primary-antibodies/akt-antibody/9272

27. Phospho-Akt (Ser473) (D9E) XP® Rabbit mAb #4060
The species: H M R Hm Mk Dm Z B
Application: WB/IP/IHC/IF/F
Website: https://www.cellsignal.com/products/primary-antibodies/phospho-akt-ser473-d9e-xp-rabbit-mab/4060

28. Phospho-Akt (Thr308) Antibody #9275
The species: H M R
Application: WB/IP
Website: https://www.cellsignal.com/products/primary-antibodies/phospho-akt-thr308-antibody/9275

29. p70 S6 Kinase Antibody #9202
The species: H M R Mk
Application: WB/IP
Website: https://www.cellsignal.com/products/primary-antibodies/p70-s6-kinase-antibody/9202

30. Na, K-ATPase Antibody #3010

The species: H M R Mk Z
Application: WB
Website: https://www.cellsignal.com/products/primary-antibodies/na-k-atpase-antibody/3010

31. Rabbit anti-dystrophin Spring Bioscience #E2660
The species: H M R Mk Z
Application: WB/IF
Reference: Xu et al. CRISPR-mediated Genome Editing Restores Dystrophin Expression and Function in mdx Mice. Mol Ther. 2016, 24(3):564-9.

32. Mouse anti-MyHC-lla (SC-71) Developmental Studies Hybridoma Bank #AB_2147165
The species: All
Application: WB/IF/IHC
Website: https://www.citeab.com/antibodies/149925-sc-71-myosin-heavy-chain-type-iia?des=8b752d8a96c4c263

33. Mouse anti-MyHC-llb ((10F5) Developmental Studies Hybridoma Bank #AB_1157896
The species: All
Application: WB/IF/IHC
Website: https://www.citeab.com/antibodies/150969-10f5-myosin-heavy-chain-type-iib?des=8d25f35312e6d839

34. Mouse anti-MyHC-I (BA-D5) Developmental Studies Hybridoma Bank #AB_2235587
The species: All
Application: WB/IF/IHC
Website: https://www.citeab.com/antibodies/149923-ba-d5-myosin-heavy-chain-type-i?des=d77a68e968050b90

35. Mouse Anti-Caveolin 3 BD #610420
The species: Mouse/Rabbit/Rat
Application: WB/IF/IHC/IP
Website: https://www.citeab.com/antibodies/3289253-610420-bd-transduction-laboratories-purified-mouse?des=66ecab936f88c3e1

36. mouse anti-puromycin clone 12D10 Sigma-Aldrich #MABE343
The species: Human/Mouse /Rat/others
Application: WB/IF/IHC/IP/others
Website: https://www.citeab.com/antibodies/1473291-mabe343-anti-puromycin-antibody-clone-12d10?des=674e1f5af4a50487

37. Atg16L1 (D6D5) Rabbit mAb #8089
The species: H M R
Application: WB/IP/IF
Website: https://www.cellsignal.com/products/primary-antibodies/atg16l1-d6d5-rabbit-mab/8089

38. Atg5 (D5F5U) Rabbit mAb #12994
The species: H M R
Application: WB/IP
Website: https://www.cellsignal.com/products/primary-antibodies/atg5-d5f5u-rabbit-mab/12994

39. Atg9A (D4O9D) Rabbit mAb #13509
The species: H M R MK
Application: WB/IP
Website: https://www.cellsignal.com/products/primary-antibodies/atg9a-d4o9d-rabbit-mab/13509

40. PI3 Kinase Class III (D9A5) Rabbit mAb #4263
The species: H M R MK
Application: WB/IP
Website: https://www.cellsignal.com/products/primary-antibodies/pi3-kinase-class-iii-d9a5-rabbit-mab/4263

41. Rabbit anti-VPS15 Cell Signaling Technology #14580
The species: H M R
Application: WB
Website: https://www.cellsignal.com/products/primary-antibodies/pik3r4-antibody/14580

42. Rab7 (D95F2) XP® Rabbit mAb #9367
The species: H M R MK
Application: WB/IP/IF
Website: https://www.cellsignal.com/products/primary-antibodies/rab7-d95f2-xp-rabbit-mab/9367

43. Rabbit anti-LAMP1 Cell Signaling Technology #9091
The species: H M R MK
Application: WB/IP/IF/others
Website: https://www.citeab.com/antibodies/654354-9091-lamp1-d2d11-xp-rabbit-mab?des=afd7775978421334

44. Rabbit anti-STX17 Proteintech #17815-1-AP
The species: Human/Mouse/Rat
Application: WB/IP/IF/others
Website: https://www.citeab.com/antibodies/979948-17815-1-ap-syntaxin-17-antibody?des=84fcd855a1520d8f

45. mTOR (7C10) Rabbit mAb #2983
The species: H M R MK
Application: WB/IP/IF/others
Website: https://www.cellsignal.com/products/primary-antibodies/mtor-7c10-rabbit-mab/2983

46. Phospho-mTOR (Ser2448) Antibody #2971
The species: H M R MK
Application: WB
Website: https://www.cellsignal.com/products/primary-antibodies/phospho-mtor-ser2448-antibody/2971

47. Rabbit anti-SNAP29 Proteintech #12704-1-AP
The species: Human/Mouse/Rat
Application: WB/IF/others
Website: https://www.citeab.com/antibodies/977219-12704-1-ap-snap29-antibody?des=dbf4f52801416e4b

48. Rabbit anti-phospho-TFEB(Ser142) Sigma-Aldrich #ABE1971
The species: Human/Mouse
Application: WB
Website: https://www.citeab.com/antibodies/3290306-abe1971-anti-phospho-tfeb-ser142-antibody?des=25dd918969e89f52

49 TFEB Antibody #4240
The species: Human/Mouse
Application: WB/IP/others
Website: https://www.citeab.com/antibodies/654216-4240-tfeb-antibody?des=25dd918969e89f52

50 Mouse anti-Flag M2 Sigma-Aldrich #F3165
The species: Human/Mouse/others
Application: WB/IP/others
Website: https://www.citeab.com/antibodies/575237-f3165-monoclonal-anti-flag-r-m2-antibody-produced-in?des=3ebc5d360d32053a

51 Rabbit anti-VAMP8 Cell Signaling Technology #13060
The species: Human/Mouse
Application: WB
Website: https://www.citeab.com/antibodies/1472745-13060-vamp8-antibody?des=10fc5770bd19a2fa

Eukaryotic cell lines

Policy information about cell lines and Sex and Gender in Research

Cell line source(s)	Cosl and HEK293 cell lines were obtained from the American Type Culture Collection (ATCC).
Authentication	The Cosl and HEK293 were not authenticated.
Mycoplasma contamination	The Cosl and HEK293 cells were not tested for mycoplasma contamination.
Commonly misidentified lines (See ICLAC register)	To the best of our knowledge, no misidentified cell lines have been used in this study.

Animals and other research organisms

Policy information about studies involving animals; ARRIVE guidelines recommended for reporting animal research, and Sex and Gender in Research

Laboratory animals	C57BL/6N mice were purchased from The Jackson Laboratory (Bar Harbor, ME). The BVES-KO mice (C57BL/6NBvestml. l(KOMP)Vlcg/MbpMmucd) were obtained from Mutant Mouse Resource & Research Centers, UC Davis and maintained in the barrier facility at the Ohio State University. The mice from 1 to 26 months of age were used for experiments. All mice were maintained under standard conditions of constant temperature (72±4°F), humidity (relative, 30–70%), in a specific pathogen-free facility and exposed to a 12-h light/dark cycle.
Wild animals	This study did not involve wild animals.
Reporting on sex	This study used both male and female mice. The sex of the animals were identified.
Field-collected samples	This study did not involve samples collected from the field.
Ethics oversight	The animal experiments were ethically reviewed by the Animal Care, Use and Review Committee of the Ohio State University and carried out in accordance with animal use guidelines.

Note that full information on the approval of the study protocol must also be provided in the manuscript.
